# Calcinosis Universalis Secondary to Silicone Injections in a Patient With HIV and Chronic Kidney Disease: A Case Report of Silicone-Induced Hypercalcemia

**DOI:** 10.7759/cureus.68325

**Published:** 2024-08-31

**Authors:** Micaela L Mayer, Rachel Reise, Juan D Sarmiento

**Affiliations:** 1 Internal Medicine, Universidad de Buenos Aires, Buenos Aires, ARG; 2 University of Florida College of Pharmacy, University of Florida Health, Gainesville, USA; 3 Division of Hospital Medicine, University of Florida Health, Gainesville, USA

**Keywords:** recurrent nephrolithiasis, chronic kidney disease (ckd), diffuse soft tissue calcinosis, hiv aids, severe hypercalcemia

## Abstract

Medical literature has long reported evidence of complications associated with cosmetic procedures, including silicone injections. Recent years have seen an increase in case reports involving hypercalcemia resulting from these injections. A common current hypothesis for the development of hypercalcemia associated with silicone injections is granulomatous inflammation against a foreign body.

This report aimed to describe the case of a 44-year-old African American male with human immunodeficiency virus (HIV) and chronic kidney disease (CKD) who presented to our hospital and was diagnosed with calcinosis universalis secondary to a history of silicone injections, as well as to present a literature review of silicone-induced hypercalcemia.

This was a case report (n=1) from a large academic medical center for which the patient, who first presented in May 2023, had two inpatient admissions and two outpatient visits before being lost to follow-up. Relevant images, laboratory results, and treatments were included.

The patient's history was significant for HIV, hypertension, CKD, recurrent nephrolithiasis, and tobacco use disorder. Physical examination was positive for flank pain while labs were significant for Na 137 mmol/L, K 2.7 mmol/L, blood urea nitrogen (BUN) 28 mg/dL, creatinine 3.72 mg/dL, calcium 13.4 mg/dL, hemoglobin 9.3 g/dL, white blood cell count 6,700 u/L and platelet count 105,000 u/L. Renal ultrasound revealed bilateral nephrolithiasis and left-sided hydronephrosis. Computerized tomography (CT) upon admission showed hyperlucid deposits in the bilateral gluteal area. Initial management included intravenous (IV) fluids and one dose of IV pamidronate, which resulted in reduced calcium levels during the admission. Subsequent management included outpatient follow-up with endocrinology during which denosumab was prescribed. This case had similar findings to other reports in the literature detailing silicone-induced hypercalcemia, which also reported abnormal imaging or nephrolithiasis, low-normal parathyroid hormone (PTH), normal 25-hydroxyvitamin D, and elevated 1,25-dihydroxyvitamin D.

Silicone injection-induced hypercalcemia should be considered as a differential diagnosis in patients presenting with otherwise unexplained elevated serum calcium and a history of past cosmetic procedures. If suspected, the use of imaging techniques (e.g. positron emission tomography (PET) scans or MRI) may help ascertain the diagnosis. Further research is needed to determine the most appropriate therapies for complex patients such as those with immunodeficiency or renal disease.

## Introduction

Injectable silicone has been used during the last decades as a filler for cosmetic procedures targeting different locations, including the face, lips, hips, and buttocks. Since the 1980s, medical literature has reported complications secondary to these injections [1].

During recent years, there has been an increasing number of case reports published describing silicone injections deposited in different parts of the body and inducing hypercalcemia, commonly described as serum calcium levels above 10 mg/dL. While the exact mechanism is unknown, various hypotheses have been proposed, including granulomatous inflammation against a foreign body [2].

We present a case of severe hypercalcemia in a male patient with human immunodeficiency virus (HIV) infection and associated acute kidney injury (AKI) on chronic kidney disease (CKD). Hypercalcemia first developed in our patient 20 years after silicone injections. The manifestation of silicone-induced hypercalcemia many years later often presents a challenging and delayed diagnosis. This case aims to contribute to the existing literature to deepen the understanding of this rare complication and its implications for patients with similar presentation, which can be crucial for clinical management and therapeutic decision-making. To our knowledge, there is only one other similar case known of severe hypercalcemia causing calcinosis universalis secondary to silicone injections in a patient with HIV [3].

## Case presentation

A 44-year-old African American male patient presented to our hospital in May 2023. His past medical history was significant for HIV, hypertension, CKD, recurrent nephrolithiasis, and tobacco use disorder. He presented to our emergency department (ED) with non-specific complaints that included headache, slurred speech, gait disturbance, and confusion. The review of systems was significant for flank pain. The blood pressure (BP) was 207/117 mmHg, heart rate (HR) 80 bpm, respiratory rate (RR) 16 pm and oxygen saturation 96%. Upon physical examination, the patient was in moderate distress, there was no presence of neck masses, and the abdomen was non-tender with no peritoneal signs, but flank pain was positive. Labs were significant for Na 137 mmol/L, K 2.7mmol/L, blood urea nitrogen (BUN) 28 mg/dL, creatinine 3.72 mg/dL, calcium 13.4 mg/dL, hemoglobin 9.3 g/dL, white blood cell count 6,700 u/L and platelet count 105,000 u/L. Urinalysis revealed pyuria and positive leukocyte esterase with no significant bacteriuria. Renal ultrasound revealed bilateral nephrolithiasis and left-sided hydronephrosis.

The patient was admitted for the management of hypertensive urgency and workup of hypercalcemia, nephrolithiasis, and AKI. Antihypertensives were started, and the patient received management for hypercalcemia with intravenous (IV) fluids and one dose of IV pamidronate. Calcium levels slowly improved during admission. Secondary workup for hypercalcemia was obtained as detailed in Table 1. PTH during the first admission was inappropriately normal for the degree of hypercalcemia. 

**Table 1:** Laboratory values of metabolic workup for hypercalcemia 1,25 OH-Vit D = 1,25-dihydroxy vitamin D; 25 OH-Vit D = 25-hydroxyvitamin D; PTH = parathyroid hormone; N/A = not applicable (lab was not collected at this visit)

ENCOUNTER	Date	Calcium	Ionized Calcium	Phosphorus	1,25OH Vit D	25OH Vit D	PTH
First admission	May-23	13.4 mg/dl	1.77 mg/dl	1.8 mg/dl	69.2 ng/ml	19 ng/ml	37 pg/ml
Second admission	Jun-23	12.4 mg/dl	1.59 mg/dl	3 mg/dl	62.4 ng/ml	30 ng/ml	20 pg/ml
After the first dose of denosumab	Aug-23	8.9 mg/dl	N/A	2 mg/dl	N/A	N/A	N/A
Last visit to the clinic	Oct-23	13.3 mg/dl	N/A	3.2 mg/dl	N/A	N/A	N/A

**Figure 1:**
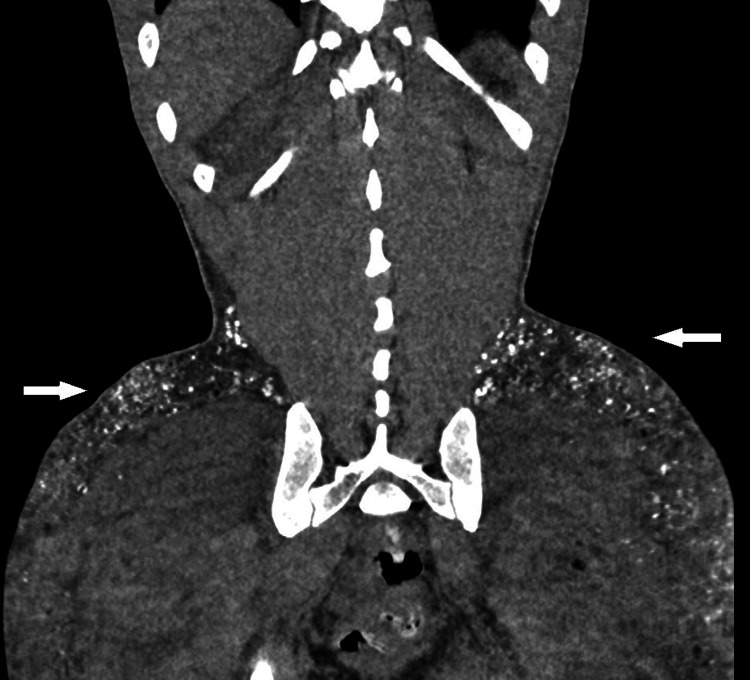
Coronal view of a computed tomography image from the chest to the proximal thighs, showing diffuse calcification around the gluteus and thighs bilaterally

**Figure 2:**
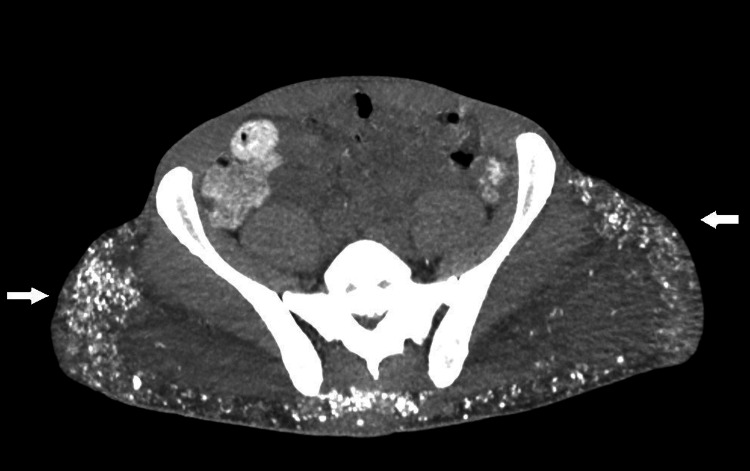
Axial view of a computed tomography image of the pelvis area revealing widespread calcification in the gluteal area bilaterally

Of note on the abdomen computerized tomography (CT) upon admission, there was evidence of hyperlucid deposits in the bilateral gluteal area (Figures 1, 2). Extensive calcium deposits of the bilateral gluteal region and proximal thigh were described by radiology. The patient also had evidence of significant renal calculi burden bilaterally. Upon further questioning, the patient mentioned silicone injections 20 years ago. Considering parathyroid hormone (PTH) was at a low normal level despite severe hypercalcemia (PTH: 37 pg/ml), primary hyperparathyroidism was a consideration. However, considering elevated 1,25-dihydroxy vitamin D, exogenous production of 1,25 dihydroxy vitamin D by silicone deposits was also considered in the differential diagnosis. The patient was discharged with instructions to follow up in the clinic. His calcium at the time of discharge was 11.5 mg/dL.

The patient was readmitted in June, one month following the first admission. He was advised to come to the ED after a clinic visit, where his serum calcium was found to be 12.4 mg/dL. The patient was asymptomatic at the time of this admission. Repeated hypercalcemia workup was repeated during this encounter as outlined in Table 1. The management of his hypercalcemia consisted of IV hydration and calcitonin. Additional labs and imaging studies also included angiotensin-converting enzyme (ACE) levels and parathyroid hormone-related protein (PTHrp). The PTHrp result was normal and ACE levels were 83 U/L (Ref. range 16-85 U/L). PTH had decreased compared to the first admission.

After seeing that his calcium levels did not improve during the last admission, during this admission nephrologists suggested starting denosumab. Due to his chronic kidney disease, bisphosphonates were not considered to be the most appropriate therapy for this patient. The patient left against medical advice two days after admission, with persistent uncontrolled hypercalcemia (calcium level of 13.0 mg/dL).

The patient was able to follow up as an outpatient one week after discharge with the endocrinology clinic where denosumab was prescribed; after insurance authorization, the patient was administered the first dose around mid-July. The patient also completed a positron emission tomography (PET) scan as an outpatient per endocrinology recommendation, which revealed extensive subcutaneous calcification in the fat of the bilateral flanks and proximal thighs, consistent with calcinosis universalis with a max standardized uptake value (SUV) of 3.04. Numerous large renal calculi were also observed on this scan.

In August, he presented to the urology clinic for follow-up regarding nephrolithiasis. His calcium level during that visit was 8.9 mg/dL, revealing an adequate response to the first dose of denosumab. He underwent left percutaneous nephrolithotomy and stent placement. The stent was removed one month later. He had a residual stone, as well as stones affecting the right kidney, but he declined to continue with more treatments.

**Figure 3:**
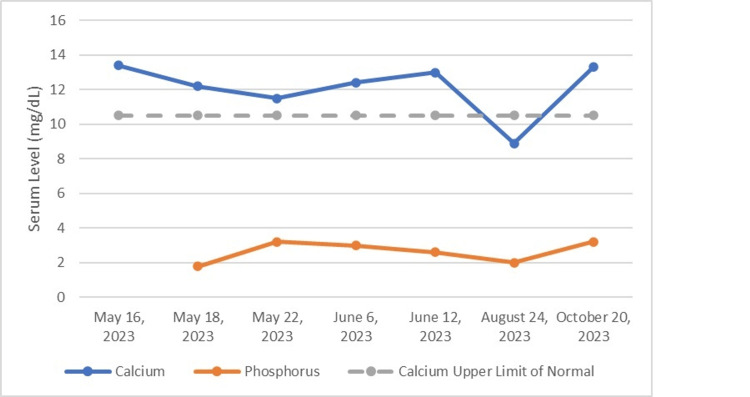
Serum calcium and phosphorus levels of the patient over time

The last blood work for this patient was obtained during one of his final visits in October. At this time his calcium level was 13.3 mg/dL, which had increased compared to the last reference obtained in August. The patient was supposed to receive his second dose of denosumab in January 2024 but was then lost to follow-up. Figure 3 shows the variation in the patient’s calcium and phosphorous levels from the first admission to the last follow-up.

## Discussion

Silicone is known to be a rare cause of hypercalcemia, often years after its administration as part of cosmetic procedures. In 1964, Winer et al. described three biopsy-proven cases of granuloma formation after silicone injection, naming them "siliconomas" [4]. They observed siliconomas with a polarized microscope, noting the presence of crystals in the tissue. This constitutes the initial evidence of silicone's role in causing this phenomenon.

The exact mechanism of this granulomatous response is not completely understood but is thought to be similar to what happens in sarcoidosis-induced granulomas. The initial process for granuloma formation requires the collaboration of macrophages, dendritic cells, and lymphocytes, but the role of the immune system in maintaining the granuloma once it is formed is not well understood [5]. What is well-known is that vitamin D plays an important role in sarcoidosis. The excessive production of active vitamin D within the body in this condition is thought to be responsible for the development of dysregulations in calcium metabolism. Under normal circumstances, vitamin D is produced exclusively in the kidneys but in sarcoidosis, there is an extrarenal source [6]. A study performed in 1983 in patients with sarcoidosis demonstrated that alveolar macrophages were responsible for the conversion of inactive vitamin D (25OH vitamin D) to active vitamin D [7]. This is due to the presence of 1α-hydroxylase activity [6,8]. Once the macrophages are activated, as they do not have down‐regulation of 1α‐hydroxylase in response to high levels of vitamin D, the absence of feedback leads to a dysregulation in its production. The consequent increase in vitamin D is responsible for unregulated calcium absorption, leading to hypercalcemia [6].

Kozeny et al. reported the first case of silicone-induced hypercalcemia in 1984 [9]. Since then, an increasing number of cases associating silicone as a causal factor for hypercalcemia have been subsequently reported. A recent systematic review in 2018 by Tachamo et al. described 23 cases of patients (mostly female) who had received silicone, polymethylmethacrylate, or paraffin oil, and years later developed hypercalcemia [10]. Injections of silicone in the breasts were the most common procedure in these cases, followed by injections in the buttocks. In this meta-analysis, hypercalcemia developed within a mean time of 7.96 (± 7.19) years from the initial injections in comparison to our case, which was 20 years. In general, 1,25-OH vitamin D levels were elevated while 25-OH vitamin D and PTH were low. As seen in Table 1, our patient’s 1,25-OH vitamin D levels were slightly elevated while PTH and 25-OH vitamin D were normal. Later, in 2019, Dangol presented 15 cases of silicone-induced hypercalcemia [11]. Similar to the review by Tachamo et al., most of the patients reported were female [10].

The literature has extensively detailed the link between silicone injections and kidney and urological disease, pointing out that acute kidney injury (AKI) and nephrolithiasis are frequently observed. Based on the review by Tachamo et al., renal failure was identified as the prevailing complication in these patients, leading to death in two of them [10]. Additionally, in 2018, Hamadeh et al. documented a case concerning a 35-year-old male bodybuilder who had undergone multiple silicone injections and presented with hypercalcemia, acute kidney injury, and nephrocalcinosis [12]. Fareen et al. documented another case in which a 31-year-old transgender woman was admitted to the hospital with renal failure and siliconomas [13].

As mentioned above, nephrolithiasis was also observed in some cases as a complication of silicone injections. In 2021, Albert S Ha et al. reported a case of a 35-year-old female with silicone-induced hypercalcemia who presented with bilateral obstructing ureteral calculi, resembling aspects of our case [14]. The following year, Yedla et al. presented another case involving nephrolithiasis, in which a 48-year-old female with severe hypercalcemia presented to the hospital after having undergone buttock silicone injections 10 years prior [2]. Laboratory values closely resembled those seen in our case: low-normal PTH, normal 25-hydroxyvitamin D, and elevated 1,25-dihydroxyvitamin D. 

Diagnostic tools like CT scans or PET fluorodeoxyglucose (FDG) are useful in the evaluation of granulomatous diseases like sarcoidosis or amyloidosis and in the diagnostic and prognostic evaluation of malignancies [15]. PET FDG has also proven to be useful in the evaluation of unexplained hypercalcemia. Nowadays, PET FDG plays an important role in the diagnosis of siliconomas, which can be seen as hypermetabolic nodules in the study images. It is not infrequent to find these siliconomas as incidental findings many years after patients received silicone injections [16]. Our patient had significant calcium deposits in the PET scan and repeated CT scan (Figures 1, 2) during his recurrent admissions, with evidence of calcinosis universalis that compromised fat of the bilateral flanks and proximal thighs. Similar imaging findings have been documented in many cases described within the literature. In the case series by Dangol et al in 2019, a case was presented of a 67-year-old woman with hypercalcemia, in which CT findings revealed calcinosis universalis bilaterally affecting the musculature of the upper thighs [11]. In the case reported by Yedla et al., a PET/CT scan was performed and showed metabolic activity in the buttocks and thighs bilaterally [2]. Then, Shirvani et al. documented the case of a 33-year-old male with firm, well-defined lesions detected in the subcutaneous tissue of his anterior pectoralis, triceps, and biceps bilaterally [17]. These lesions were indicative of sclerosing granulomas, a diagnosis that was confirmed by magnetic resonance imaging (MRI). Pando et al. presented the case of a 40-year-old transgender female patient with HIV and CKD stage 3b and hypercalcemia, who had previously received silicone injection [3]. A non-contrast CT scan performed on this patient revealed widespread thickening of the skin in the breasts and buttocks, along with scattered punctate calcifications. In another case of a 39-year-old female published by Huf et al., an MRI of the pelvis exhibited multiple foreign body granulomas penetrating the depth of superficial muscle and scattered throughout the gluteal region, lower anterior pelvis, and proximal thighs [18]. These observations allow us to conclude that image studies play an important role in detecting these lesions.

In the above-mentioned cases, calcitriol levels were usually elevated as compared to our patient in whom calcitriol was just above the upper limit of normal, which portrays the different mechanisms of actions not involving Vitamin D directly and contributing to the development of hypercalcemia in our patient. It is also worth mentioning that during the initial workup of our patient, PTH was not significantly suppressed, which prompted the differential diagnosis. Although primary on the second admission, PTH was adequately suppressed for the level of hypercalcemia, ruling out this disease. This makes us consider that hypercalcemia in our case was most likely multifactorial, and other non-calcitriol-related mechanisms can contribute to this common outcome in patients with silicone injections.

Currently, there is no definitive treatment for silicone-induced hypercalcemia. Many of the above-mentioned case reports used strategies like hydration, bisphosphonates, and systemic steroids as the most common management. In our patient, due to his chronic kidney disease, bisphosphonates were not an appropriate first-line therapy, and for this reason, the managing endocrinologist considered denosumab for his outpatient therapy. Our patient had some improvement in his hypercalcemia from the first dose but was lost to follow-up for repeated doses. This may be considered a reasonable alternative in patients with similar clinical profiles accompanied by CKD, although more research is needed.

## Conclusions

Silicone injection-induced hypercalcemia should be considered a differential diagnosis in patients presenting with elevated serum calcium and a history of past cosmetic procedures when admitted to the hospital. If suspected, utilizing imaging techniques, such as PET scans and MRI, may help ascertain the diagnosis. As evidenced by the numerous reports in the literature on long-term effects that have been observed, it is crucial to implement public health campaigns to educate people about the potential health risks associated with these procedures such as renal, urological, and metabolic complications. Ongoing efforts are needed to elucidate the available therapies that could most benefit these patients, as well as the role of new therapies used for general hypercalcemia in this specific setting.
